# Morphotropic Phase Boundary Enhanced Photocatalysis in Sm Doped BiFeO_3_

**DOI:** 10.3390/molecules27207029

**Published:** 2022-10-18

**Authors:** Farid F. Orudzhev, Nariman M.-R. Alikhanov, Shikhgasan M. Ramazanov, Dinara S. Sobola, Rabadanov Kh. Murtazali, Etibar H. Ismailov, Rashid D. Gasimov, Akif Sh. Aliev, Ştefan Ţălu

**Affiliations:** 1 REC Smart Materials and Biomedical Applications, Immanuel Kant Baltic Federal University, 236041 Kaliningrad, Russia; 2 Department of Inorganic Chemistry and Chemical Ecology, Dagestan State University, St. M. Gadjieva 43-a, Dagestan Republic, 367015 Makhachkala, Russia; 3 Amirkhanov Institute of Physics of Dagestan Federal Research Center, Russian Academy of Sciences, 367003 Makhachkala, Russia; 4 Department of Physics, Faculty of Electrical Engineering and Communication, Brno University of Technology, Technicka 10, 616 00 Brno, Czech Republic; 5 Central European Institute of Technology, Purkyňova 656/123, 612 00 Brno, Czech Republic; 6 Institute of Catalysis and Inorganic Chemistry, National Academy of Sciences of Azerbaijan, H. Cavid Ave. 113, AZ 1143 Baku, Azerbaijan; 7 Institute of Radiation Problems, Azerbaijan National Academy of Sciences, 9 B. Vahabzade Str., AZ 1143 Baku, Azerbaijan; 8 Directorate of Research, Development and Innovation Management (DMCDI), Technical University of Cluj-Napoca, Constantin Daicoviciu Street, No. 15, 400020 Cluj-Napoca, Romania

**Keywords:** BFO, bismuth ferrite, morphotropic phase boundary, nanocomposite, photocatalysis, polarization, Sm

## Abstract

This paper presents the results of the synthesis of samarium-doped bismuth ferrite (BFO) nanoparticles by the solution combustion method. The dependence of BFO properties on the amount of the samarium (Sm) in the composition was studied. The synthesized nanocomposites were characterized by scanning electron microscopy SEM), X-ray diffractometry (XRD), Raman, Electron Diffuse Reflectance Spectroscopy (EDRS) and Electron Magnetic Resonance (EMR). The photocatalytic (PC) measurements showed the absence of a strict correlation between the PC activity and the crystallite size and band gap. An increase in the PC activity of BFO samples with 10 and 15% doping was observed and it was concluded that in controlling the PC properties in doped BFO, the processes of interfacial polarization at the boundaries of the morphotropic phase transition are of decisive importance. It was supposed that the internal electric field formed at these boundaries contributes to the efficient separation of photogenerated charge carriers.

## 1. Introduction

The topic of the photocatalytic (PC) decomposition of organic water pollutants continues to be one of the hottest topics. Perovskite materials and ferrites have generated strong research interest in recent decades as they are among the most promising materials for photocatalytic degradation of pollutants as well as hydrogen generation due to their unique properties and the possibility of their controlled customization [1,2,3,4,5,6]. It has recently been shown that the combination of perovskite materials with ferrites and an S-type heterojunction made it possible to significantly enhance the photocatalytic properties [7]. Bismuth ferrite (BiFeO_3_, BFO) is a unique material that combines the properties of perovskite materials and ferrites [8]. One of the important advantages of this material is the smaller band gap (2.2 eV) compared to the traditional TiO_2_ photocatalyst [9,10]. This is important for improving the efficiency of solar energy use. BFO has been synthesized using various methods, and is used in the decomposition and mineralization of pollutants [11,12]. One of the synthesis methods that allow the synthesis of phase-pure BFO with a highly developed surface is solution combustion [13,14,15,16]. In our previous work, pure-phase BFO was synthesized by solution combustion method and its photocatalytic activity was studied [17]. The photocatalytic activity of BFO is highly dependent on particle size, morphology, crystallinity, and surface chemistry. Doping is known to be one of the main strategies for controlling material properties [18]. Doping of BFO, especially with rare earth elements, shows a significant improvement in the photocatalytic properties of the material [19,20,21,22]. In all of the mentioned studies, an increase in the photocatalytic activity of a doped material is reduced to the appearance of new energy states in the band gap, a narrowing of the band gap, and a decrease in particle size, leading to more efficient separation of photogenerated charge carriers. However, it has been shown that, depending on the amount of dopant, in BFO doped with rare earth elements Sm, Nd, Ho, and Er, a structural phase transition from the rhombohedral phase to the orthorhombic phase can be observed [23,24,25]. At the same time, at certain doping values, the rhombohedral and orthorhombic phases coexist, forming a morphotropic phase boundary (MPB), leading to a sharp increase in the permittivity and piezoelectric coefficient [26]. This indicates that the presence of MPB in the material increases the role of polarization processes, which form locally internal electric fields, which will affect the behavior of photogenerated charge carriers. It should also be noted that the unique properties of BFO and materials based on it open up broad prospects for application in various magnetoelectric devices, spintronics, sensor technology, and magnetic memory [27,28,29,30].

In this work, the phase composition, morphology, structure, and catalytic properties in the photodegradation of MO of synthesized BFO and doped with samarium BFO as a function of samarium concentration are presented, and the formation of a morphotropic phase boundary in these systems and its role in photocatalytic activity are discussed.

## 2. Results

The first step in the synthesis process is the formation of a coordination complex of metal ions with glycine since the latter is a complexing agent:

Bi(NO_3_)_3_·5H_2_O + Fe(NO_3_)_3_·9H_2_O + Sm(NO_3_)_3_·6H_2_O +NH_2_CH_2_COOH →  Bi/Sm[Fe(NH_2_CH_2_COO)](NO_3_)_3_·H_2_O + NO_2_ + H_2_O          (1)

Nitrate ions in the complex itself provide the combustion process with the necessary oxygen. The complex, when heated, decomposes to oxides of bismuth, iron, and samarium:

Bi/Sm[Fe(NH_2_CH_2_COO)](NO_3_)_3_·H_2_O → Bi_2_O_3_ + Sm_2_O_3_ + Fe_2_O_3_ + NO_2_ + H_2_O + CO_2_ (2)

Bi, Fe and Sm oxides combine to form Bi_1−x_Sm_x_FeO_3_.

Bi_2_O_3_ + Fe_2_O_3_ + Sm_2_O_3_ → Bi_1−x_Sm_x_FeO_3_ (3)

During self-combustion, non-stoichiometric reactions between Bi_2_O_3_, Fe_2_O_3_, and Sm_2_O_3_ occur in some parts due to temperature unevenness throughout the gel. Thus, in this case, BiFeO_3_ is always accompanied by the formation of a small number of side phases, such as Bi_2_Fe_4_O_9_, Bi_25_FeO_39_, etc. Another important reason for the formation of secondary phases is due to the narrow temperature range in which BiFeO_3_ crystallizes. Below is the complete stoichiometric reaction. In the case of combustion of the glycine-nitrate complex, mainly N_2_, CO_2_, and H_2_O are emitted in the form of gaseous products:

6Bi(NO_3_)_3_·5H_2_O + 6Fe(NO_3_)_3_·9H_2_O + 6Sm(NO_3_)_3_·6H_2_O + 32NH_2_CH_2_COOH = 6(Bi/Sm)FeO_3_ + 43N_2_ + 200H_2_O + 64CO_2_                   (4)

**Figure 1:**
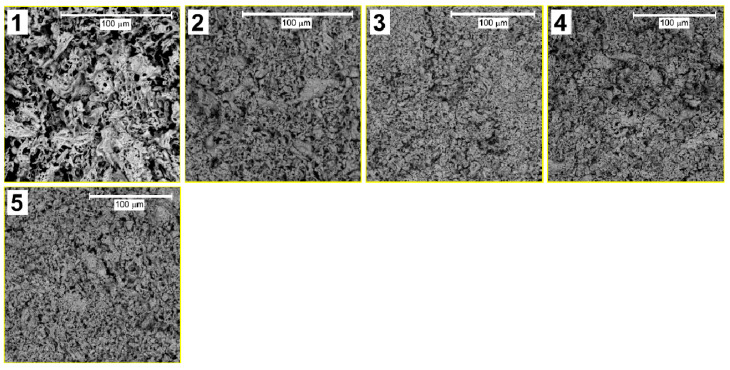
SEM images of Bi_1−x_Sm_x_FeO_3_ (*x* = 0.00—1, 0.05—2, 0.10—3, 0.15—4, 0.20—5).

**Figure 2:**
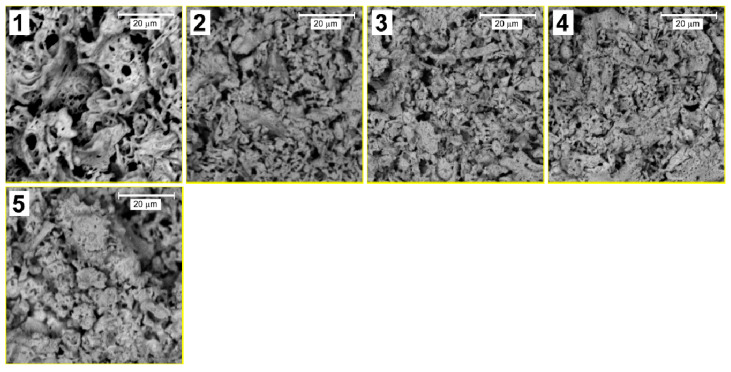
SEM images of Bi_1−x_Sm_x_FeO_3_ (*x* = 0.00—1, 0.05—2, 0.10—3, 0.15—4, 0.20—5).

To study the morphology of the obtained samples, images were obtained by a scanning electron microscope (Figure 1 and Figure 2).

As can be seen from Figure 1, the samples have a highly porous structure. The interstices have an irregular shape, and their size varies in the submicron range. Cavities in this case are created due to the rapid release of many combustion gases. The nanoparticles are in agglomerates that are densely packed and have a uniform morphology. This morphology of the samples makes it almost impossible to analyze the shape or size of the grains. Higher resolution images (Figure 2) show that with increasing Sm concentration, the surface becomes less homogeneous and more developed, which indicates the presence of point structural defects on the surface caused by the substitution of Bi atoms (1.03 Å) in the BiFeO_3_ crystal structure into Sm atoms (0.958 Å), the ionic radius of which is smaller [31].

Surface inhomogeneity plays a significant role in processes on the surface. Foreign atoms form dipole moments on the surface, which can differ greatly in magnitude and direction from the core [32,33]. We believe that this effect should further increase the photocatalytic activity of the material since photocatalysis is a surface process. The sizes of agglomerates upon alloying from 5 to 10% decrease noticeably and, at the same time, porosity increases. With a further increase in the Sm concentration from 15 to 20%, particle enlargement and the formation of massive agglomerates up to 10 µm in size are observed.

This effect can, in our opinion, be explained by two factors:

(1) As mentioned above, the ionic radius of Sm is smaller than that of Bi. Therefore, an increase in % substitution will reduce the size of crystallites, and, consequently, nanoparticles. As the particle size decreases, the contribution of van der Waals interactions between individual particles becomes significant. Due to these interactions, individual nanoparticles are agglomerated.
(2) When Bi atoms are replaced by Sm atoms, a crystal lattice stress is created, and in this case, an excess dipole moment is accumulated on the surface of the nanoparticles, and the surface has an excess of free energy. However, as is known, from a thermodynamic point of view, the material will be the most stable when the Gibbs free energy is minimal. Therefore, under equilibrium conditions, the shape of the crystal tends to be one in which the value of the surface energy of the crystal is minimal. Since one of the main ways to reduce the surface energy of nanoparticles is to reduce the total surface area, agglomeration is observed.

**Figure 3:**
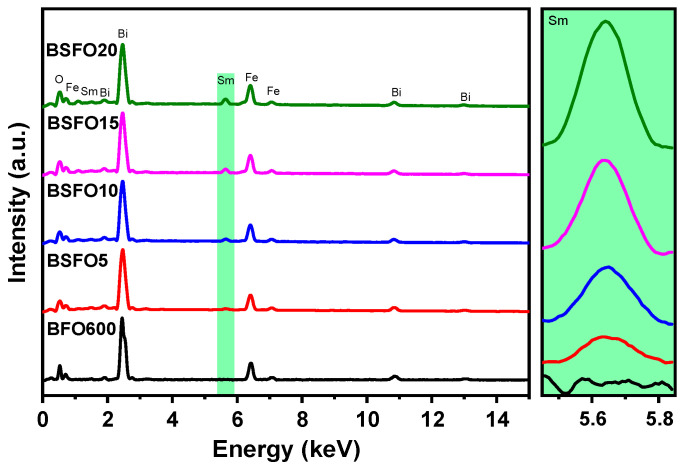
Energy-dispersive spectra of Bi_1−x_Sm_x_FeO_3_ samples (*x* = 0.00, 0.05, 0.10, 0.15, 0.20).

To confirm the presence of Sm atoms in the synthesized materials and the absence of additional impurities, we performed the elemental analysis of the samples. The analysis was carried out by a scanning electron microscope over the total area of the images at 250 × 250 µm. Figure 3 shows the energy-dispersive spectra of the samples. It can be seen from the spectra that the samples contain only Bi, Fe, O, and Sm atoms. The inset to the right of Figure 3 shows that the Sm concentration increases logically from 5 to 20% doping. To confirm the assumptions associated with structural changes in the sample during doping and the data of scanning electron microscopy, X-ray diffraction, and X-ray phase analysis were carried out.

**Figure 4:**
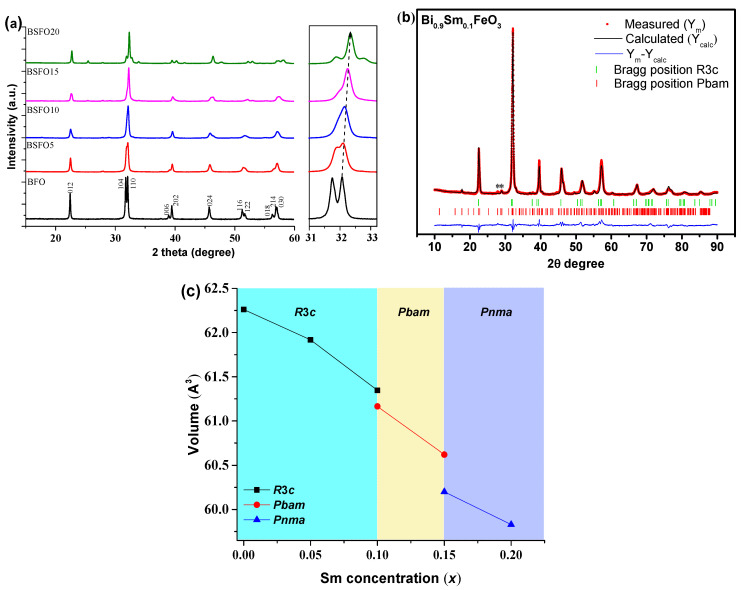
(**a**) Results of diffraction analysis of Bi_1−x_Sm_x_FeO_3_ samples (*x* = 0.00, 0.05, 0.10, 0.15, 0.20), (**b**) refined X-ray pattern by the Rietveld method of the Bi_0.9_Sm_0.1_FeO_3_ sample; (**c**) unit cell volume depending on *x*.

Figure 4 shows X-ray diffraction patterns of nanoparticle samples containing various Sm doping concentrations. The XRD patterns of the Bi_1−x_Sm_x_FeO_3_ (*x* = 0.00, 0.05, 0.10, 0.15, 0.20) powders calcined at 600 °C for 30 min are shown in Figure 4. Phase analysis showed that in almost all samples, a small amount of impurities (less than 1% of phases), such as Bi_2_Fe_4_O_9_ and Bi_25_FeO_39_, can be observed [34,35,36]. In the case of doped BSFO formulations in the concentration range 0.05 ≤ *x* ≤ 0.20, the diffraction peaks are shifted towards higher values, which indicates lattice distortions (Figure 4a) because of the smaller ionic radius of samarium.

The compositions of Bi_1−x_Sm_x_FeO_3_ (*x* = 0.00; 0.05) crystallize in a rhombohedral structure with the space symmetry group *R*3*c*. When the samarium concentration is increased to 10%, the crystal structure of the BSFO is described by a two-phase model: along with the rhombohedral phase *R*3*c*, the antipolar orthorhombic *Pbam* phase is formed in the ratio ~1:1 (Table 1).

The *Pbam* space group suggests the antipolar nature of the displacement of ions in the A- and B-positions of perovskite [22,26]. It should be noted that in a few works [37,38,39,40,41], it is reported that a 10% substitution of Bi^3+^ for Sm^3+^ does not lead to any structural changes that are possible at a higher concentration. However, refinement of the XRD patterns of this composition by the Rietveld method indicates the presence of a new phase of *Pbam*. Sample BSFO15 (Bi_0.85_Sm_0,15_FeO_3_) is also described by a two-phase model however in this case the *R3c* phase is absent and a new *Pnma* phase appears. In the case of BSFO20 (Bi_0.8_Sm_0.2_FeO_3_), the structure is completely described by a single-phase model with the *Pnma* space group. The average crystallite size, calculated by Scherrer’s formula, is 56, 40, 35, 34 and 30 nm for *x* = 0.00, 0.05, 0.10, 0.15 and 0.20, respectively. An increase in Sm content would result in a unit cell volume contraction, because the ionic radius of Sm^3+^ is smaller than that of Bi^3+^ [42] (Figure 3). With increasing samarium fraction, the size of crystallites decreases.

**Figure 5:**
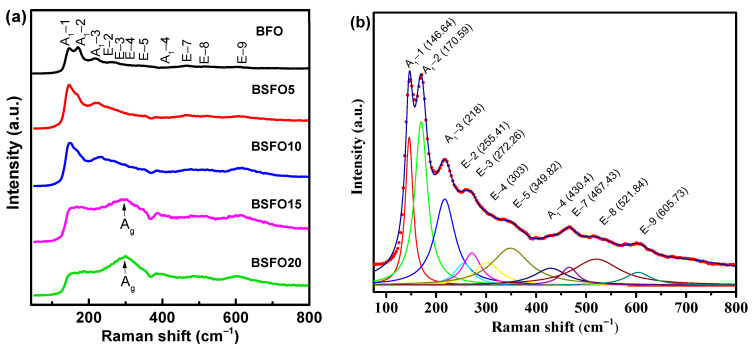
(**a**) Raman spectra of Bi_1−x_Sm_x_FeO_3_ (*x* = 0.00, 0.05, 0.10, 0.15, 0.20). (**b**) Lorentz-fitted Raman spectrum of pure BFO.

Raman spectra for BSFO, taken at room temperature, are shown in Figure 5.

For rhombohedral BFO with space group *R*3*c*, thirteen (4A_1_ + 9E) active modes are expected [43,44]. For composition *x* = 0.00, eleven Raman modes were observed in total: 146.64 cm^−1^ (A_1_-1), 170.59 cm^−1^ (A_1_-2), 218 cm^−1^ (A_1_-3), 430.4 cm^−1^ (A_1_-4), 255.41 cm^−1^ (E-2), 272.26 cm^−1^ (E-3), 303 cm^−1^ (E-4), 349.82 cm^−1^ (E-5), 467.43 cm^−1^ (E-7), 521.84 cm^−1^ (E-8), 605.73 cm^−1^ (E-9) (see Figure 5b). At 10% Sm substitution, the most intense mode (A_1_-1) shifts to 149 cm^−1^. At a Sm concentration of more than 10%, the Raman spectra undergo significant changes, indicating structural distortions. The mode (149 cm^−1^) disappears completely when the Sm concentration reaches *x* ≥ 0.15. The broadening of the Raman modes with an increase in the Sm concentration corresponds to a decrease in the average crystallite size, which is in agreement with the XRD data. For *x* = 0.15–0.20, a broad peak is observed at 300 cm^−1^. This is due to the A_g_ mode, which arises due to the vibration of Sm–O bonds in an orthorhombic unit cell [45]. The 260 cm^−1^ mode (E-mode) is associated with the Fe-O covalent bond and the corresponding Fe-O-Fe angle. Modes exceeding 600 cm^−1^ refer to second-order Raman scattering, which is associated with electron–phonon interaction in BiFeO_3_.

**Figure 6:**
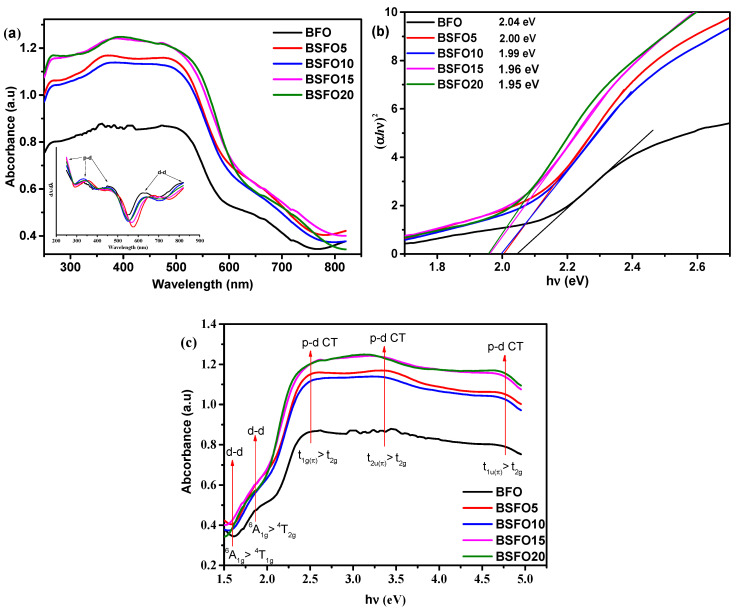
(**a**) UV–Vis absorption spectra; (**b**) plot of (*αhν*)^2^ versus energy (*hν*) to determine the band gap energy. Lines indicate the direct extrapolation (DE) technique; (**c**) absorption vs. photon energy curves for Bi_1−x_Sm_x_FeO_3_ (*x* = 0.00, 0.05, 0.10, 0.15, 0.20).

To study the optical characteristics of light absorption of photocatalysts Bi_1−x_Sm_x_FeO_3_ (*x* = 0.00, 0.05, 0.10, 0.15, 0.20), diffuse reflectance spectra (DRS) of UV-Visible light were obtained. The results are shown in Figure 6.

All samples exhibit strong light absorption in both the UV and visible region, demonstrating that the synthesized samples can exhibit photocatalytic activity under UV-Visible illumination. The edge of the absorption band of BFO nanoparticles is located near 600 nm, as pointed out by other authors [46]. Compared to pure BFO, samples doped with Sm show increased absorption capacity, especially in the visible light region. The intensity of absorption in this case gradually increases, with an increase in the content of the dopant. The spectra show five transitions, which are in the range from 1.5 to 4.5 eV. Similar results were also obtained by other authors [47,48]. From the DRS BFO spectrum, as shown in Figure 6, the shoulder centered at 1.6 and 2 eV corresponds to the ^6^A_1g_ → ^4^T_1g_ and ^6^A_1g_ → ^4^T_2g_ transitions, respectively, which arise due to d-d excitation of the crystal field of Fe^3+^ ions in BFO. These forbidden excitations appear due to the spin-orbit coupling, which weakens the spin selection rule [49,50]. Above 2 eV, absorption increases significantly, and peaks appear at ~2.5, ~3.3, and 4.5 eV, which can be attributed to p–d charge transfer (CT) excitations [49]. The peak of the CT transition centered at ~2.5 eV can be unambiguously attributed to the dipole forbidden t_1g(__π)_→t_2g_ p-d CT transition like other ferrites with FeO_6_ centers [51]. Intense bands near 3.3 and 4.5 eV are assigned to dipole allowed t_2u(__π)_→t_2g_ and t_1u(__π)_→t_2g_ p-d CT transitions in octahedral FeO_6_ centers, respectively [49]. For BFO compounds with samarium substitution, a slight redshift is observed at d-d and p-d CT transitions. This indicates that substitution in BFO increases the internal “chemical pressure”, which results from changes in the local environment in the FeO_6_ octahedra. This confirms the data of XRD spectra refinement by the Rietveld method, shown in Table 1, which indicates the decrease in the volume of the unit cell.

**Figure 7:**
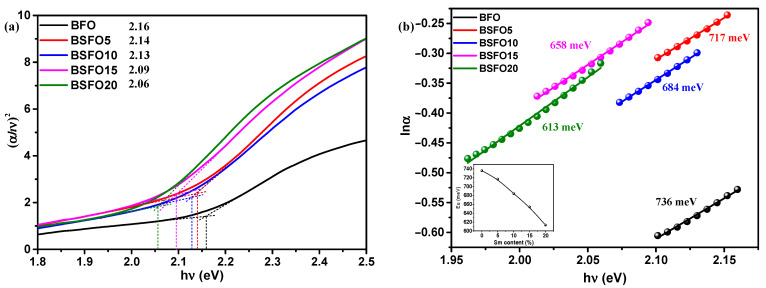
(**a**) Plot of (*αhν*)^2^ versus energy (*hν*) to determine the band gap energy. Lines indicate the proper extrapolation (PE) technique; (**b**) ln*α* versus *hν* plots to determine the Urbach energy for Bi_1−x_Sm_x_FeO_3_ (*x* = 0.00, 0.05, 0.10, 0.15, 0.20).

The optical band gap was calculated from the Kubelka–Munk plot (Figure 6b) plotted in the coordinates F((*αhν*)^2^) – (*hν*)), because BFO is a direct-gap semiconductor. The band gaps were 2.05, 2.00, 1.99, 1.96, and 1.95 eV for pure BFO, 5%, 10%, 15%, and 20% Sm doping, respectively. In this case, we applied the most common method for determining the band gap, the direct extrapolation (DE) method. The band gap is estimated by fitting a straight line to the linear part of the absorption spectrum. However, in [52], it is reported that this method is inaccurate. Proceeding from this, we estimated the value of the band gap using the “proper extrapolation” (PE) method, in which the point of intersection of linear approximations of the absorption edge and the baseline is taken as the band gap value. The results are shown in Figure 7a.

From a comparison of the two techniques for determining the band gap, it can be seen that the direct extrapolation gives slightly underestimated values. However, the nature of dependence remains the same, and the band gap decreases with increasing samarium content. A change in the band gap value can be caused by various reasons, such as structural distortions arising when Bi atoms are replaced by Sm, a change in the size of crystallites, and the presence of oxygen vacancies [53]. The creation of oxygen vacancies can be caused by valence nonstoichiometry of iron ions. In [8], we demonstrated that pure BFO synthesized by solution combustion is characterized by the presence of iron in +2 and +3 oxidation states. In addition, Sm doping creates localized states in the band gap near the valence band, which can also be regarded as a decrease in the band gap. The density of localized electronic states in synthesized systems can be estimated from the Urbach characteristic energy (E_u_), which can be calculated from the rate of exponential decay of the “tail” in the absorption spectrum by equation α = α_o_exp(hν/E_u_). The width of the Urbach tail is an indicator of the disorder in the material. Thus, Eu can be calculated from lnα and hν as the inverse slope of the curve (Figure 7b). The calculated value of Eu near the edge is 736 meV for the original BFO sample and decreases monotonically to 613 meV for the BSFO20 sample (see inset in Figure 7b). A decrease in E_u_ values indicates that Sm doping reduces the number of oxygen vacancies. All data on optical measurements are given in Table 2.

**Figure 8:**
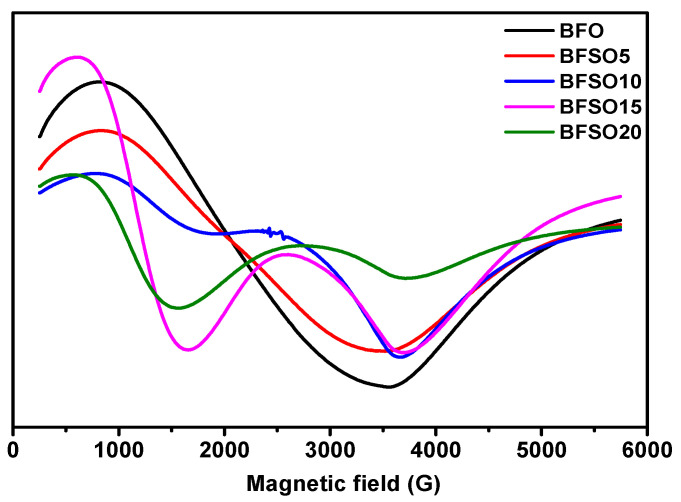
EMR spectra of Bi_1−x_Sm_x_FeO_3_ (*x* = 0.00, 0.05, 0.10, 0.15, 0.20).

Electron magnetic resonance (EMR) is a useful tool for studying spin dynamics in ferromagnetism and antiferromagnetism [54]. Measurement of the magnetic moment provides information on local magnetic properties, the nature of spin–spin interactions, the distribution of the internal field, and spin-lattice correlations. In Figure 8 EMR spectra of BFO and Bi_1−x_Sm_x_FeO_3_ with different concentration of samarium are presented.

As shown in Figure 8, Sm doping strongly affects the character of the EMR spectra. EMR spectra can be divided into two resonant regions, which are associated with the presence of various types of defects and magnetic anisotropy: the low-field (LF) and high-field (HF) resonance regions. As a rule, magnetic dipole interactions and superexchange interactions between magnetic ions through oxygen ions are two important factors that determine the values of the g factor and the width of the resonance line. Superexchange interactions usually increase when the distance between magnetic ions and oxygen ions decreases, and the corresponding bond angles are close to 180. Strong dipole interactions give a large resonant linewidth and g value, while strong superexchange interactions give a relatively small linewidth and g value.

**Figure 9:**
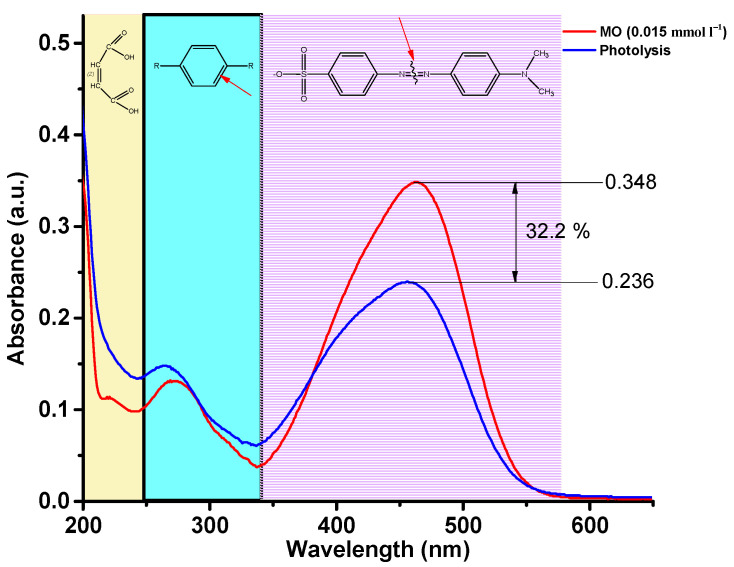
Absorption spectra of MO solution over time during photolysis.

The EMR spectrum of pure BFO powder gives an almost isotropic signal with a g factor of 3.3280 and a width of ∆B = 277.0 mT. The EMR spectrum of BSFO5 is also a wide signal; however, it is slightly asymmetric and consists of a superposition of two signals with a total effective g-factor of 3.2847 and a width of ∆B = 271.0. The presence of a high-field isotropic signal is because the magnetic moments in the sample are randomly oriented with respect to the external magnetic field. For the samples with a high content of samarium (10, 15, 20%), EMR spectra are observed, consisting of a superposition of two signals, the intensity ratio of which depends on the Sm content in the samples. One of the signals is in the low-field resonance arm with g = 6.4934, which is characteristic of magnetically isolated high-spin Fe^3+^ ions (S = 5/2) in a medium with low symmetry (tetragonally coordinated Fe^3+^ ions), corresponding to Fe^3+^–Vo•• defective dipoles. Another signal is a high-field resonance with g = 2.118 associated with the presence of Fe^3+^ ions in the octahedral field because of superexchange interactions in the lattice [55], which can also be related to resonant absorption in the cycloidal spin structure and defects caused by uncompensated spins. The splitting of the resonant signal is obviously associated with a change in the magnetic medium for unpaired electrons in Fe ions, as well as the possible presence of a secondary magnetic phase. All obtained samples were studied in the process of photocatalytic decomposition of MO. To exclude the effect of MO photolysis under the action of UV-Vis irradiation, a blank experiment was carried out. Figure 9 shows the absorption spectra of the initial MO solution and the MO solution after 180 min of irradiation.

**Figure 10:**
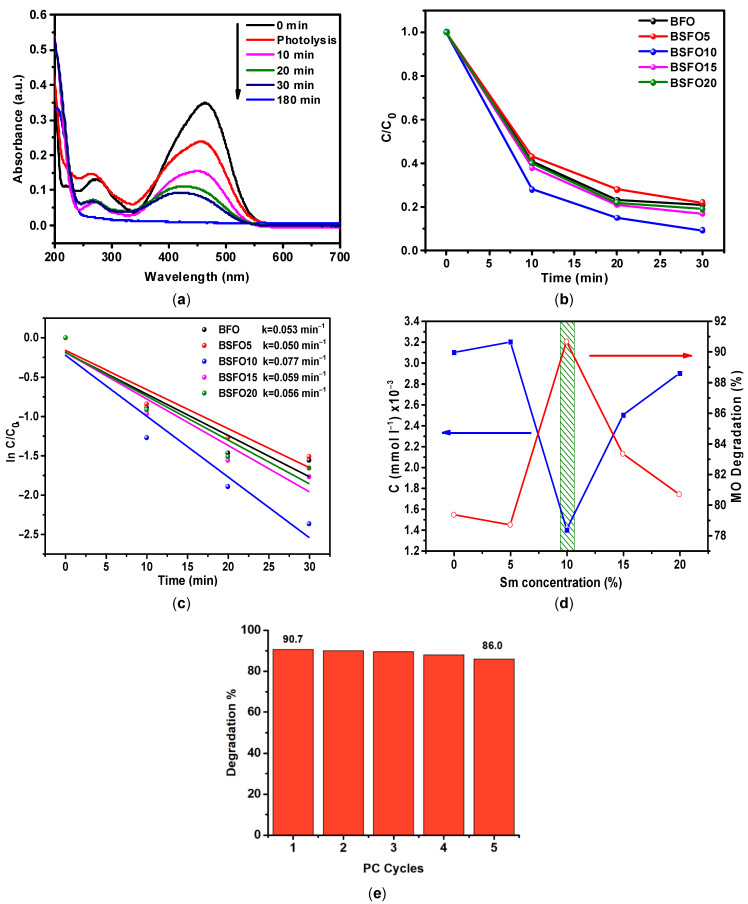
(**a**) The absorption spectra of MO solution over time in a photocatalytic experiment for BSFO5. (**b**) C/C_0_ curve of MO degradation. (**c**) Pseudo-first order kinetics of the photocatalytic reactions. (**d**) Comparative photocatalytic activity of Bi_1−x_SmxFeO_3_ (*x* = 0.00, 0.05, 0.10, 0.15, 0.20). (**e**) Photocatalytic degradation of MO with the BSFO10 sample for five cycles.

Three regions can be clearly distinguished on the spectrum of the initial MO solution, 340–580 nm, characterizing the presence of –N=N– bonds, 240–340 nm, characterizing the presence of aromatic rings, and 200–240 nm, corresponding to the absorption of unsaturated aliphatic acids. As can be seen, after three hours of irradiation, the concentration of the MO solution decreased by 32.2%. At the same time, it should be noted that the peaks at 200–240 and 240–340 nm increase, which indicates that the –N=N– bond of MO molecules is broken, and many intermediate aromatic and aliphatic molecules are formed. Figure 10 shows the absorption spectra of the MO solution over time in a photocatalytic experiment using BSFO5 as a photocatalyst.

It can be seen from the data that 78.2% of the dye decomposes during the first 30 min of the process. It is also seen that the peak at 240–340 nm, which characterizes the presence of aromatic compounds, decreases significantly, but the peak associated with the presence of unsaturated aliphatic compounds in the solution increases strongly. This indicates that highly oxidizing radicals are generated in the solution during the photocatalytic process, which effectively decomposes aromatic organic compounds. On the spectrum of the MO solution after 180 min, there is only a peak characterizing the presence of aliphatic compounds. Similar measurements were carried out with samples doped with Sm 10, 15, 20%, as well as pure BiFeO_3_. The data are presented in Figure 10b as a C/C_0_ ratio plot. It can be observed from the obtained results presented in Figure 10c that the process corresponds to pseudo first-order kinetics. For easier comprehension, the data are presented in Figure 10d. It can be seen from the data that the Bi/Sm substitution increases the photocatalytic activity of BFO. In this case, the sample with 10% Sm with a substitution of more than 90% in 30 min of the process exhibits the highest photocatalytic activity. It can also be seen that the photocatalytic activity of pure BFO and 5% Sm of the substituted sample is almost the same. The results once again confirm that the concentration of Sm^3+^ substitution can affect the photocatalytic activity of BFO and that there is an optimal doping concentration of Sm^3+^ ions. The stability and reusability of a photocatalyst is an important parameter for practical applications. To evaluate the stability and reusability of BSFO10 sample, photocatalyst was recycled for five runs, as depicted in Figure 10e. After five successive runs, the degradation efficiency could be largely maintained, indicating good stability.

The formation of highly active oxidants is closely related to the energy of the conduction band (CB) and the valence band (VB) of the semiconductor. The CB and VB potentials of BiFeO_3_ were determined using Mulliken’s theory of electronegativity.

*E_VB_* =*X* − *E^e^* + 0.5*E_BG_* (5)

*E_CB_* = *E_VB_* − *E_BG_* (6)

where *E_VB_* is the valence band potential, *X* is the electronegativity of the semiconductor, χ is the electronegativity of the element, *E^e^* is the standard potential of the hydrogen electrode (~4.5 eV), *E_BG_* is the optical band gap, *E_CB_* is the conduction band potential. 

The electronegativity of the semiconductor was calculated according to Equation (7):

(7) $$X_{\left(BiFeO_{3}\right)}={\left(\chi {\left(Bi\right)}^{1}\cdot \chi {\left(Fe\right)}^{1}\cdot \chi {\left(O\right)}^{3}\right)}^{\frac{1}{1+1+3}}$$

and the electronegativity of a neutral atom according to Mulliken is the arithmetic mean of the affinity of an atom for an electron (*E_EA_*) and the first ionization potential (*E_IP_*):

(8) $$\chi =\frac{E_{IP}+E_{EA}}{2}$$

**Figure 11:**
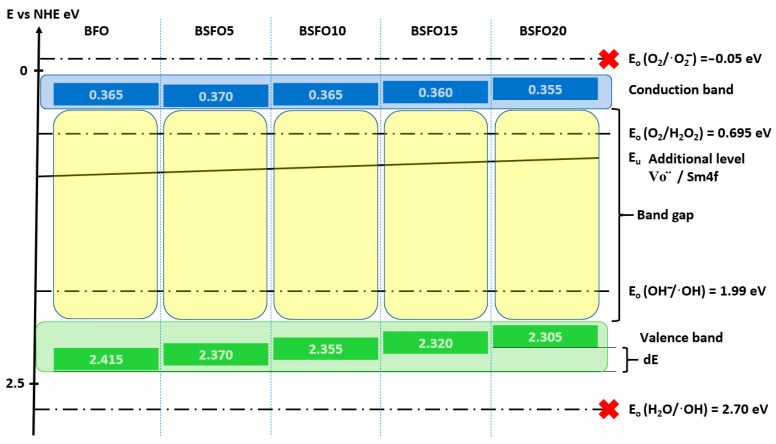
Relative band position and possible photocatalysis mechanism for Bi_1−x_Sm_x_FeO_3_ (*x* = 0.00, 0.05, 0.10, 0.15, 0.20) under UV-Vis light illumination.

Since the values of ionization potentials and electron affinities for most atoms are given in tables, Mulliken’s electronegativity becomes a parameter with absolute electronegativity values based only on measurable physical quantities. In a sense, Mulliken’s electronegativity is the electrochemical potential of an electron in a neutral atom. The layout of the energy levels is shown in Figure 11.

Calculations showed that doping does not affect the energy position of the CB remaining at a level of ≈0.4 eV, while the top of the VB shifts monotonically from 2.415 eV for BFO to 2.305 eV for BSFO20. It is also seen that the position of the Urbach energy, caused by additional energy levels in the BG, shifts slightly to the bottom of the VB. This confirms the conclusion that the proportion of oxygen vacancies decreases with increasing doping. Based on these results, a possible mechanism for the photocatalytic reaction can be proposed. When BiFeO_3_ is irradiated with light with an energy comparable to or greater than the BG, an electron–hole pair (h^+^/e^−^) is generated (9).

BFO + hv → h^+^ + e^−^ (9)

The electrons in the BG of BiFeO_3_ are not able to reduce O_2_ to ·O_2_^−^, since the BG potential of all samples (≈0.4 eV vs. NHE) is more positive than the standard redox potential *E*^0^ (O_2_/·O_2_^−^) (−0.18 eV vs. NHE). However, the band gap potential of BiFeO_3_ is more negative than the standard redox potential *E*^0^ (O_2_/H_2_O_2_) (0.695 eV vs. NHE), so oxygen adsorbed on the semiconductor surface will react with two electrons to form H_2_O_2_ (10), which subsequently, when interacting with an electron, forms ·OH (11).

O_2_ + 2e^−^ + 2H^+^ → H_2_O_2_ (10)

H_2_O_2_ + e^−^ →·OH + OH^−^ (11)

The VB of BiFeO_3_ (≈2.4 eV relative to NHE) is more positive than the standard redox potential *E*^0^ (·OH/OH^−^) (1.99 eV relative to NHE), which indicates that holes localized in the VB can oxidize adsorbed OH^−^ with the formation of ·OH (12).

OH_ad_ + h^+^ → ·OH (12)

Thus, we can conclude that the main route of MO decomposition is oxidation by hydroxyl radicals. The discrepancies in the photocatalytic activity of Bi_1−x_Sm_x_FeO_3_ (*x* = 0.00, 0.05, 0.10, 0.15, 0.20) can be explained by the following factors:

1. Firstly, Sm doping reduces the band gap and increases optical absorption in the UV and visible light region. This means that since more charge carriers will be generated when irradiated with UV-visible light, the efficiency of photodegradation increases. However, in our case, with doping, the width of the band gap systematically decreases; however, the photocatalytic activity increases only up to *x* = 0.10, and then a noticeable decrease is observed. Therefore, other reasons need to be considered.
2. Secondly, the Sm^3+^ substitution causes lattice deformation and, by changing the local environment of atoms, modifies the electronic structure. Since rare earth elements are known to be good electron acceptors [56], they will act as traps to capture excited electrons, which probably facilitates the separation of photogenerated electron–hole pairs and prolongs the lifetime of charge carriers [57], which ultimately enhances photocatalytic activity. However, excess amounts of Sm^3+^ dopant can act as recombination centers in BFO, resulting in low PC activity at high doping %.
3. Thirdly, the rhombohedral *R*3*c* phase in BFO and BSFO5 is noncentrosymmetric (polar), has ferroelectric properties, and exhibits spontaneous polarization. The orthorhombic *Pbam* phase with antipolar Bi-O and Sm-O dipole moments is antiferroelectric, exhibiting weak spontaneous polarization, which is compensated within the unit cell. The orthorhombic *Pnma* phase in BSFO15 and BSFO20 is centrosymmetric, paraelectric, and nonpolar. In the absence of an internal electric field in these samples, photogenerated electrons and holes easily recombine, which should significantly reduce photocatalytic performance compared to BFO, BSFO5, and BSFO10.

**Figure 12:**
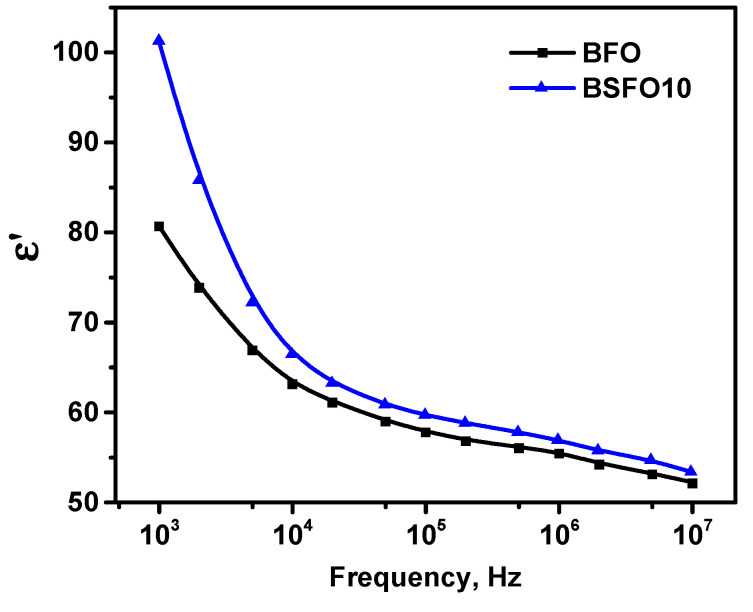
Frequency dependence of permittivity for pure BFO and BSFO10.

Thus, we confirm that the emerging internal electric field in polar noncentrosymmetric structures can separate photogenerated electrons and holes, reducing charge recombination. However, the fact that the PC activity of BSFO10, in which polar–antipolar phases coexist, is higher than that of BFO with a purely polar phase, seems illogical. To understand this effect, we performed dielectric measurements. It is known that material properties determined by polarization change, such as dielectric constant, can be enhanced in phase transition regions where there is a significant change in polarization [58]. Figure 12 shows the frequency dependences of the permittivity for pure BFO and BSFO10.

For both samples, the permittivity decreases with increasing frequency, which can be explained by the effect of space charge relaxation based on polarization processes. The improvement in permittivity at *x* = 0.10 can be explained by the replacement of Bi^3+^ by smaller Sm^3+^, which provides an increase in the dipole moment. On the other hand, the contribution to the permittivity is made by space charges accumulating at the grain boundaries. Since at *x* = 0.10 the grain size decreases compared to pure BiFeO_3_, this leads to an increase in the grain boundaries. In addition to intergrain boundaries, the charges accumulated at the interphase boundaries of the MPB inside the grains contribute to the enhancement of the permittivity. Table 3 shows the comparative characteristics of the catalytic activity of some doped BFO catalysts.

From the presented data, it can be seen that our material is significantly superior in terms of its photocatalyst properties to photocatalysts of a similar composition from the works of other authors.

## 3. Materials and Methods

### 3.1. Synthesis of Bi_1−x_Sm_x_FeO_3_ Nanoparticles

Nanoparticles of BiFeO_3_ were obtained using a new method of synthesis [8]. Analytically pure bismuth (Bi(NO_3_)_3_·5H_2_O), iron (Fe(NO_3_)_3_·9H_2_O), samarium (Sm(NO_3_)_3_·6H_2_O) nitrates and glycine were taken according to the stoichiometric ratio. Glycine played the role of fuels, which provided a platform for redox reactions between reagents during combustion. Metal nitrates are hygroscopic, and they tend to form a suspension mixture when mixed with glycine. The mixture was heated to ~300 °C for dehydration and initiation of the combustion process. Since metal nitrates also play the role of oxidizing agents, the combustion process can occur efficiently using the oxygen contained in the reactants themselves. As a result of the combustion process, ash was formed, and the yellow vapor was released. Then, the sample was heat-treated at temperatures of 600 °C for 30 min. The stoichiometry of the combustion reaction was calculated using the concepts of propellant chemistry [17], which shows a simple method for calculating the redox valences of the mixture components. According to these concepts, metals, carbon, and hydrogen are considered reducing elements with corresponding metal valences, +4 for carbon and +1 for hydrogen. Oxygen is considered to be an oxidizing agent with a valence of −2, and the valence of nitrogen is 0. The combustion reaction is highly exothermic, and the maximum combustion temperature is achieved when the equivalence ratio (*φ_e_* is the oxidizer/fuel relation) is equal to unity. The equivalence relation was found according to [64]. In this work, we synthesized Bi_1−x_Sm_x_FeO_3_ precisely at *φ*_e_ = 1. The resulting Bi_1−x_Sm_x_FeO_3_ (*x* = 0, 0.05, 0.10, 0.15, 0.20) samples will be referred to below as BFO.

### 3.2. Characterizations

X-ray diffraction studies were performed using an Empyrean PANalytical X-ray diffractometer using the radiation of a copper anode with a nickel filter. Data processing was performed using the High Score Plus application program, included in the instrument software, and the diffraction database PDF-2. The morphology of the obtained samples was studied using an ASPEX EXpress scanning electron microscope equipped with an energy dispersive X-ray spectrometer (EDS). The morphology was studied in the mode of detection of secondary electrons. Raman spectra were measured by Ntegra Spectra (λ = 532 nm laser). The electron paramagnetic resonance spectra were recorded at room temperature using an EMXmicro spectrometer, Bruker, Germany, with an operating frequency of 9.8 GHz. Diffuse reflection spectra (DRS) in the coordinates F(R) = *f*(λ, nm), where F(R) is the Kubelka–Munk function, were performed on a Shimadzu UV-3600 spectrophotometer with an integrating sphere LISR-3100. To determine the width of the band gap of the obtained materials, we used the method of constructing the Kubelka–Munk curve by converting the optical absorption spectra using the values of the absorption coefficient (K) and the photon energy (*hv*) using the wavelengths: (*hv* = 1240/λ). The graph was plotted in coordinates (K*hv*)^1/2^ to (*hv*) and by extrapolating the curve to the zero value of the absorption coefficient, it was possible to find the energy value of the band gap.

### 3.3. Photocatalytic Measurements

The photocatalytic characteristics of the catalysts were evaluated by photodegradation of methyl orange (MO) in an aqueous solution (0.015 mmol/L). Photocatalytic experiments were carried out in a 100 mL quartz cell. As the light source, the 150 W high-pressure Xenon lamp with 410 nm cutoff filter was used. A constant cell temperature of 26 °C was maintained by air ventilation and monitored with a thermometer. For the photocatalytic reaction, 25 mg of the photocatalyst was added to 50 mL of an aqueous solution of MO (0.015 mmol). Before turning on the light, the cell was placed in the dark for 30 min to achieve adsorption equilibrium. Before the start of the experiment, the suspension was subjected to ultrasonic treatment to degas the photocatalyst. The whole process was carried out with magnetic stirring. Sampling (5 mL) was carried out every 10 min, large particles of the nanopowders were subjected to magnetic separation using a powerful neodymium magnet to prevent loss of the photocatalyst, and then centrifuged at 14,000 rpm for 3 min on an MR23i (Thermo Fisher Scientific, JOUAN, France) high-speed refrigeration centrifuge for deposition of ultrafine nanoparticles. The concentration of MO was measured using a Beckman Coulter DU730 series UV/Vis spectrophotometer at a constant temperature of 26 °C. After measurement, the solution was poured back into the cell and the process continued. For comparison, we also tested the MO solution under similar conditions, without a photocatalyst. The concentration of MO was determined by the Bouguer–Lambert–Beer law.

## 4. Conclusions

BFO nanoparticles doped with samarium in various amounts from 5 to 20% were synthesized using the solution combustion method. It was shown that an increase in the content of samarium leads to structural distortions. The sizes of crystallites and the unit cell volume decrease. As doping increases, the band gap also decreases. An analysis of the crystal structure showed that doping leads to phase transitions with the formation of morphotropic phase boundaries. Thus, at 10% doping, the polar rhombohedral *R*3*c* and antipolar orthorhombic *Pbam* phases coexist. At 15% doping, the system already consists of antipolar orthorhombic *Pbam* and nonpolar orthorhombic *Pnma* phases. The PC measurements showed the absence of a strict correlation between the PC activity and the crystallite size and band gap. A significant increase in the PC activity of samples with 10 and 15% doping enables us to conclude that in controlling the PC properties in doped BFO, the processes of interfacial polarization at the boundaries of the morphotropic phase transition are of decisive importance, since the internal electric field formed at these boundaries contributes to the efficient separation of photogenerated charge carriers. 

## Figures and Tables

**Table 1:** Structural parameters were obtained from the Rietveld refinement of X-ray diffraction patterns of Bi_1−x_Sm_x_FeO_3_ samples with *x* = 0.00, 0.05, 0.10, 0.15, and 0.20.

Sample	Space Group	Lattice Parameters (Å)	Volume (Å^3^)
BiFeO_3_	*R*3*c* (100%)	*a* = 5.578; *c* = 13.864;	62.26
Bi_0.95_Sm_0.05_FeO_3_	*R*3*c* (100%)	*a* = 5.574; *c* = 13.807;	61.92
Bi_0.9_Sm_0.1_FeO_3_	*R*3*c* (47.4%)	*a* = 5.565; *c* = 13.724;	61.35
	*Pbam* (52.6%)	*a* = 5.582; *b* = 11.214; *c* = 7.817;	61.16
Bi_0.85_Sm_0.15_FeO_3_	*Pbam* (84.6%)	*a* = 5.549; *b* = 11.187; *c* = 7.813;	60.62
	*Pnma* (15.4%)	*a* = 5.615; *b* = 7.843; *c* = 5.469;	60.20
Bi_0.8_Sm_0.2_FeO_3_	*Pnma* (100%)	*a* = 5.607; *b* = 7.819; *c* = 5.459;	59.83

**Table 2:** Optical properties of Bi_1−x_Sm_x_FeO_3_ samples with *x* = 0.00, 0.5, 0.10, 0.15, and 0.20.

Sample	DE Band Gap, eV	PE Band Gap, eV	Urbach Energy, meV
BiFeO_3_	*2.04*	*2.16*	*736*
Bi_0.95_Sm_0.05_FeO_3_	*2.00*	*2.14*	*717*
Bi_0.9_Sm_0.1_FeO_3_	1.99	*2.13*	*684*
Bi_0.85_Sm_0.15_FeO_3_	*1.96*	*2.09*	*658*
Bi_0.8_Sm_0.2_FeO_3_	*1.95*	*2.06*	*613*

**Table 3:** Comparison of catalytic activity between some doped BFO catalysts.

Material of Catalyst	Concentration of Catalyst	Excitation Source	Concentration of Pollutant	Time, min	Degradation Ratio, %	Ref.
**Bi_1–x_Sm_x_FeO_3_**						
***x* = 0.00** **0.01** **0.03** **0.05** **0.07** **0.10**	3 g/L	300 W Xe lamp attached with a cut-off filter (ë ≥ 420 nm)	MO solution (5 mg L^−1^, pH 6.8)	120	58.8 65.4 86.9 63.4 62.1 31.5	[59]
**Bi_1−x_Sm_x_FeO_3_**						
***x* = 0.00** **0.01** **0.03** **0.05**	0.5 g/L	Direct sunlight	15 ppm MO	180	70.3 89.6 100 79.8	[60]
**Bi_1−x_Sm_x_FeO_3_**						
***x* = 0.00** **0.05** **0.10** **0.15** **0.20**	1 g/L	300 W Xe lamp	MO aqueous solution (10^−5^ mol L^−1^)	180	72 80 72 16 14	[61]
**BFO**						
**1.5%Pd/BFO** **BSFO** **1.0%Pd/BSFO** **1.5%Pd/BSFO** **2.0%Pd/BSFO**	3 g/L	300 W Xe lamp (ë ≥ 420 nm)	MO 3 g/L	120	42 47 60 80 87 76	[62]
**Bi_1−x_Gd_x_FeO_3_**						
***x* = 0.00** **0.01** **0.03** **0.05**	3 g/L	300 W Xe lamp (ë ≥ 420 nm)	RhB 5 mg/L	270	22.3 34.2 56.8 42.1	[63]
**Bi_1–x_Sm_x_FeO_3_**						
***x* = 0.00** **0.05** **0.10** **0.15** **0.20**	0.5 g/L	150 W Xe lamp (ë ≥ 410 nm)	MO 0.015 mmol/L	30	79.3 78.7 90.7 83.3 80.7	This work

## Data Availability

The data that support the findings of this study are available from the corresponding authors upon reasonable request.

## Sample Availability

Not available.
